# Comparative Analysis of Serum (Anti)oxidative Status Parameters in Healthy Persons

**DOI:** 10.3390/ijms14036106

**Published:** 2013-03-18

**Authors:** Eugène HJM Jansen, Tatjana Ruskovska

**Affiliations:** 1 Center for Health Protection, National Institute for Public Health and the Environment, Bilthoven, The Netherlands; 2 Faculty of Medical Sciences, Goce Delcev University, Stip 2000, Macedonia; E-Mail: tatjana.ruskovska@ugd.edu.mk

**Keywords:** oxidation assays, antioxidant assays, total thiol assay, correlation

## Abstract

Five antioxidant and two oxidative stress assays were applied to serum samples of 43 healthy males. The antioxidant tests showed different inter-assay correlations. A very good correlation of 0.807 was observed between the ferric reducing ability of plasma (FRAP) and total antioxidant status (TAS) assay and also a fair correlation of 0.501 between the biological antioxidant potential (BAP) and TAS assay. There was no statistically significant correlation between the BAP and FRAP assay. The anti-oxidant assays have a high correlation with uric acid, especially the TAS (0.922) and FRAP assay (0.869). The BAP assay has a much lower and no statistically significant correlation with uric acid (0.302), which makes BAP more suitable for the antioxidant status. The total thiol assay showed no statistically significant correlation with uric acid (0.114). The total thiol assay, which is based on a completely different principle, showed a good and statistically significant correlation with the BAP assay (0.510) and also to the TAS assay, but to a lower and not significant extent (0.279) and not with the FRAP assay (−0.008). The oxy-adsorbent test (OXY) assay has no correlation with any of the other assays tested. The oxidative stress assays, reactive oxygen metabolites (ROM) and total oxidant status (TOS), based on a different principle, do not show a statistically significant correlation with the serum samples in this study. Both assays showed a negative, but not significant, correlation with the antioxidant assays. In conclusion, the ROM, TOS, BAP and TTP assays are based on different principles and will have an additional value when a combination of these assays will be applied in large-scale population studies.

Abbreviations

FRAP: ferric reducing ability of plasma
BAP: biological antioxidant potential
TAS: total antioxidant status
ABTS: 2,2′-azino-bis(3-ethylbenzotiazoline-6-sulphonic acid)
ROM: reactive oxygen metabolites
CARR U: Carratelli Units
TOS: total oxidant status
TTP: total thiols in proteins
OXY: oxy-adsorbent test

## 1. Introduction

Oxidative stress, which is defined as an imbalance between the pro-oxidant reactive species and antioxidant molecules, both endogenous and exogenous, has been associated with many non-communicable diseases, such as obesity, insulin resistance [1] and diabetes [2], atherosclerosis [3,4], autoimmune diseases [5,6], neurodegenerative diseases [7,8], chronic renal disease [9,10], different malignancies [11,12], as well as in aging [13] as a physiological process. Many clinical studies were designed to investigate different strategies for oxidative status improvement in affected individuals, such as various therapeutic approaches to the underlying disease(s) [14] or therapy with antioxidant vitamins [15].

As a result of the wide interest in the oxidative stress and antioxidants, numerous commercial test kits for oxidative stress assessment became available. These tests are dedicated primarily for use in clinical studies, although their use in experiments with animals or cell cultures, even in foods and beverages, is not excluded. Having the possibility to choose from many methods and manufacturers, it is a real challenge to choose the right combination of methods and assays for specific health research. Moreover, although time-consuming, there are still possibilities to prepare reagents in the laboratory, according to already published methods and protocols.

Therefore, the aim of our study was to make an extensive comparative analysis of the results obtained by different methods and assays for the measurement of serum oxidative stress-related parameters, both commercial and in-house. The chosen assays were tested on a group of healthy male volunteers, because the use of samples from a diseased-state can disturb the correlation, due to unexpected and unknown influences.

Therefore, our comparative study provides new insights to the past and future studies of oxidative stress research.

## 2. Methods

### 2.1. Volunteers

The 43 volunteers (all men) were selected from 50 healthy candidates for military service. The mean age of the participants was 26 ± 3 years. The participants were mentally and physically healthy, without any acute nor chronic disease. All participants had normal nutritional habits and did not report the use of any dietary supplement. All of the volunteers had an erythrocyte sedimentation rate, complete blood count, glucose, total bilirubin, asparagine aminotransferase (AST) and alanine aminotransferase (ALT) within the reference values or allowed variations: total bilirubin up to 50 μmol/L, AST and ALT up to 50 U/L and/or red blood cells up to 6 × 10^6^/mm^3^. Seven volunteers did not meet these criteria. Fasting blood samples were drawn from the antecubital vein. The blood samples were drawn in the morning at about 9:00–9:30 am, after an overnight fasting period starting at 11:00 pm. Blood was separated by centrifugation, and aliquots of serum were kept tightly closed at −70 °C until analysis. The samples were stored at −70 °C for 3 months. In a separate study, the performance of all assays were checked on human serum samples stored up to one year at −70 °C (unpublished results). All parameters turned out to be stable during this period.

All parameters were measured at the same day as a single measurement and in one analysis series, with the exception of the FRAP assay, which was measured on a different day. The study was performed and approved under the ethical guidance of Dr. Dusan Stojanovik, as documented by the Command of the Military Medical Center, Ministry of Defense of the Republic of Macedonia, and described in Document N No. 04-7/18 from the Army Mail 2990/80, Skopje, Republic of Macedonia.

### 2.2. The FRAP Assay

The measurement of the ferric reducing ability of plasma (FRAP) was done by the assay based on the method of Benzie and Strain [16], slightly modified. The method is based on the principle of the reduction of the ferric-tripyridyltriazine complex to the ferrous form, upon which an intense blue color develops, and the change of absorbance is measured at 593 nm (kinetic method). We have measured the FRAP assay in a microplate format, by the end-point approach. Briefly, 10 μL of sample and 40 μL of water were pipetted in the microplate in duplicate. After that, 200 μL of working reagent were added in each well (a: acetate buffer pH 3,6; b: FeCl_3_ solution; c: 2,4,6,-tripyridyl-s-triazine solution; 10:1:1), and the reaction mixture was incubated for exactly 8 min at 37 °C. The absorbance was measured on a Chemwell analyzer at 600 nm, against a reagent blank. Standards of 500, 1,000 and 2,000 μmol/L FeSO_4_ were used for calibration of the assay. The results of the test are expressed as μmol/L FeSO_4_. The intra-assay variation of the FRAP assay was 3.6%, as determined with two quality control samples.

### 2.3. The BAP Assay

For measurement of the biological antioxidant potential (BAP), the test kit from Diacron [17] (Grosseto, Italy) was used. The assay is based on the ability of a colored thiocyanate-derived substrate, which contains bounded Fe^3+^ ions, to decrease in absorption when Fe^3+^ ions are reduced to Fe^2+^. The absorbance is assessed photometrically by measuring the absorbance at 505 nm and calculating the amount of reduced ferric ions. The results of the test are expressed as μEq ferric ions reducing antioxidants per L of sample. The kit from Diacron was adjusted for the use on the autoanalyzer LX20-Pro from Beckman-Coulter (Woerden, The Netherlands). The intra-assay variation of the BAP assay was 1.2%, as determined with two quality control samples.

### 2.4. The TAS Assay

The total antioxidant status (TAS) was measured by the test kit from Rel Assay Diagnostics (Gaziantep, Turkey) [18]. The method is based on the reduction of colored 2,2′-azino-bis(3-ethylbenzotiazoline-6-sulphonic acid) (ABTS) radical to a colorless reduced form by the antioxidants that are present in the sample. The absorbance is measured at 660 nm. The method is calibrated with the vitamin E analog, known as trolox equivalent, and the results are expressed in mmol/L. The kit from Rel Assay was adjusted for the use on the autoanalyzer LX20-Pro from Beckman-Coulter (Woerden, The Netherlands). This TAS assay of Rel Assay is the so-called 2nd generation TAS assay of Randox with a correlation of 0.897 (*p* < 0.0001) [19]. The intra-assay variation of the TAS assay was 1.4%, as determined with two quality control samples.

### 2.5. The TTP Assay

The content of the total thiols in proteins (TTP) was measured using also the reagent kit from Rel Assay Diagnostics (Gaziantep, Turkey) [18]. The method is based on the reaction of thiols with 5,5′-dithiobis-(2-nitrobenzoic acid), upon which a colored anion is formed with an absorption peak at 412 nm. The results of the test are expressed in μmol/L. The kit from Rel Assay was adjusted for the use on the autoanalyzer LX20-Pro from Beckman-Coulter (Woerden, The Netherlands). The intra-assay variation of the TTP assay was 1.9%, as determined with two quality control samples.

### 2.6. The OXY Assay

The oxy-adsorbent test from Diacron (Grosseto, Italy) evaluates the ability of serum to oppose the massive *in vitro* oxidation, which is induced by hypochlorous acid solution. Unreacted HClO radicals further react with the chromogen solution of *N*,*N*-diethyl-p-phenylendiamine and form a colored complex, which is measured at 505 nm. The results of the test are expressed as μmol HClO/mL. Normally, 1 mL of human serum is able to adsorb at least 350 μmol of HClO. Decreased values reflect the injury of the serum barrier to oxidation. The kit from Diacron [17] was adjusted for the use on the autoanalyzer LX20-Pro from Beckman-Coulter (Woerden, The Netherlands). The intra-assay variation of the OXY assay was 3.8%, as determined with two quality control samples.

### 2.7. The Uric Acid Assay

Uric acid was measured by the method using uricase and performed on an autoanalyzer LX20-Pro from Beckman-Coulter (Woerden, The Netherlands) with a dedicated kit for uric acid. The intra-assay variation of the uric acid assay was 0.5%, as determined with three quality control samples.

### 2.8. The ROM Assay

The reactive oxygen molecules (ROM) were measured by the reagent kit dROMs from Diacron (Grosseto, Italy) [17]. The method actually measures the concentration of hydroperoxides that are present in the sample. The method is based on the principle that in an acidic buffered solution (pH = 4.8), iron ions previously bonded to serum proteins become available to catalyze the reaction of the conversion of serum hydroperoxides to alkoxyl and peroxyl radicals, which further react with chromogen *N,N*-diethyl-p-phenylendiamine. Upon oxidation, the chromogen is transformed into a red colored cation, which is measured at 505 nm. The results of the test are expressed in CARR U (Carratelli Units). Each CARR U corresponds to 0.08 mg H_2_O_2_/100 mL of the sample. The kit from Diacron was adjusted for the use on the autoanalyzer LX20-Pro from Beckman-Coulter (Woerden, The Netherlands). The intra-assay variation of the ROM assay was 3.6%, as determined with two quality control samples.

### 2.9. The TOS Assay

The total oxidant status (TOS) was measured by the test kit from Rel Assay Diagnostics (Gaziantep, Turkey) [18]. The method is based on the principle that oxidants that are present in the sample can oxidize the ferrous ions, previously bounded to a chelator, to ferric ions. Further, in an acidic medium, the ferric ions make a colored complex with a chromogen. The intensity of the color is measured at 530 nm. The assay is calibrated with H_2_O_2_, and the results of the test are expressed in μmol H_2_O_2_ Eq/L. The assay was performed in microtiter plates. The intra-assay variation of the TOS assay was 3.6%, as determined with two quality control samples.

### 2.10. Statistics

The results of all measurements are expressed as the mean ± standard deviation. The results, as well as the coefficient of correlations, were calculated by Microsoft Excel.

The statistical significance of the coefficients of correlation was assessed according to the number of subjects within the group, using a statistical table. The correlation coefficient was considered as statistically significant when it was *p* < 0.05 [20].

## 3. Results

### 3.1. Antioxidant Assays

The antioxidant assays used in this study were the FRAP, BAP, TAS, TTP and OXY assays. The first two assays are more or less based on the same principle, being the reduction of iron-complexes, followed by a change in color of the reduced iron(II)-complexes. In the 2nd generation TAS assay iron is no longer involved. The OXY assay is based on the principle of artificial oxidation by HClO_4_. The TTP assay is based on a different principle. In this assay the thiol groups in the serum, mainly attached to protein, are determined by a colorimetric substrate. In this study, uric acid was also included, because of the substantial contribution to the antioxidant status in some of the antioxidant assays.

In Table 1, the mean values of the antioxidant assay have been listed, including the inter-individual standard deviations. For BAP and TAS, the inter-individual variation is rather small, which means that changes in these antioxidant assays are relatively small. Therefore, the assay performances must be kept constant to be able to detect small changes in the antioxidant status. For FRAP, TTP and OXY, these variations were somewhat larger.

The results of the antioxidant assays have been compared, and the correlation coefficients have been listed in Table 2. The three closely related antioxidant assays, FRAP, BAP and TAS, showed a correlation behavior that is somewhat remarkable. Between TAS and FRAP, there is a very good and statistically significant correlation of 0.807. Also, between TAS and BAP, there is a statistically significant correlation of 0.501. Between FRAP and BAP, however, there is no correlation (0.097). The TTP assay showed only a good and statistically significant correlation with BAP (0.510) and not with the other antioxidant assays. The OXY assay showed no statistically significant correlation with other assays. The serum component uric acid showed very good statistically significant correlations with both TAS (0.922) and FRAP (0.869), but not with BAP (0.302).

With the two oxidation assays used in this study, all antioxidant assays showed no significant correlation.

### 3.2. Oxidation Assays

The oxidation assays measured in this study were ROM and TOS. Although both assays are based on iron-mediated mechanisms, the principle of detection is rather different. In the ROM assay, the released iron ions from transferrin initiated the formation of pre-oxidants in the serum, whereas in the TOS assay, the oxidation of the iron by oxidants is detected by a chromogenic oxidized iron complex.

The inter-individual variations for both assays are substantial (see Table 1), being 21.5% and 42.7%, for ROM and TOS, respectively. Both assay are not statistically significant correlated with each other, as is indicated in Table 2 (correlation coefficient = 0.204). Also, with the other (antioxidant assays), no significant correlation was found.

## 4. Discussion

### 4.1. Antioxidant Assays

Three closely related methods were used for determination of total antioxidants in serum samples from patients included in our study: the FRAP assay (slightly modified original method of Benzie and Strain), the BAP method (reagent kit from Diacron) and the TAS method (reagent kit from Rel Assay Diagnostics). All three methods, FRAP [21,22], BAP [23,24] and TAS [25,26], are widely recognized and used in different clinical studies of oxidative stress-related pathological conditions (only a few recent articles are cited). From these three assays, TAS correlated significantly with FRAP (0.807) and, to a lower extent, also with BAP (0.501). BAP and FRAP, however, do not correlate with each other (0.097). A possible explanation can be found in the correlation of the antioxidant assays with uric acid, one of the major serum components that determines the antioxidant status of serum. Since both TAS (0.922) and FRAP (0.869) correlate very well with uric acid in a statistically significant way, the antioxidant activities are largely determined by the contribution of uric acid. Other authors did not find a significant correlation between the outcome of FRAP and TAS [27]. Apparently, the lack of significant correlation between FRAP and BAP is probably due to the lower and statistically non-significant correlation between BAP and uric acid (0.302). In the literature, the contribution of uric acid to the antioxidant assays was found [28], and another study [19] found a good correlation of TAS with uric acid, although this could not be confirmed by others [27]. Also, for FRAP, a good correlation with uric acid was found [27].

All these results suggest that although designed to measure the same entity, that being the level of total antioxidants in the sample, the three methods could sometimes lead to different conclusions for the same set of samples. Since the BAP assay has the lowest correlation with uric acid, this antioxidant assay might have a preference for use in large-scale human studies compared to the FRAP and TAS assay. In addition the BAP assay can be automated for use with clinical autoanalyzers, as used in this study.

The TTP assay is another kind of antioxidant assay that reflects the occurrence of free thiol groups, mainly present as sulfhydryl groups as a side chain of the amino acid cysteine, both in proteins and as a free amino acid and, to a lower extent, reduced glutathione. The TTP assay showed only a good correlation with BAP. This could indicate that SH-groups contribute to the antioxidant status, as measured by BAP and not by FRAP or TAS. The BAP assay was not studied in detail in the literature as were the other antioxidant assays. But another study [28] stated that based on mechanistic reasons, thiol groups should not have a large contribution to the BAP assay [29]. In another study [30], it was found that the correlation between TAS and TTP was rather poor (0.298), and also, between TTP and uric acid, no correlation was found (0.066). Because the TTP assay can also be automated, this assay is, therefore, very suitable for large human studies and can add valuable additional information about the antioxidant status, in addition to the other antioxidant assays (BAP, FRAP and TAS).

The OXY assay shows a small and non-significant correlation with TAS and uric acid. This could indicate that the OXY assay could add an additional value in the set of assays to access the oxidation and antioxidant status of serum. The total antioxidant assays in serum, as described here, do not cover all antioxidant processes. The components present in serum that contribute to these assays included low molecular weight serum compounds (uric acid), the antioxidant vitamins, C and E, and proteins, such as albumin. These components reflect the passive antioxidant system. The antioxidant enzymes present mainly in erythrocytes, such as superoxide dismutase, catalase and enzymes responsible for a proper redox status, such as glutathione peroxidase, glutathione reductase and glutathione-s-transferases, also contribute to the overall antioxidant capacity, but do not take part in the antioxidant assays described in this study.

### 4.2. Oxidation Assays

Furthermore, we have compared two different assays for assessment of the serum oxidants: the ROM assay and the TOS assay. The oxidative stress assays, ROM and TOS, are based on a different principle; therefore, it was not surprising that no significant correlation (0.204) between these assays was observed. Both assays showed only a small negative, but non-significant, correlation with one of the antioxidant assays, FRAP, BAP, TAS, TTP and OXY. Both oxidation assays showed a large inter-individual variation, so changes in the oxidative stress status should be detected by both assays. Both the ROM and TOS assay can be automated and applied on a clinical auto-analyzer and can be used in large-scale population studies [31].

## 5. Conclusion

From the results of our study, it is clear that different results are obtained from different assays when assessing the levels of total antioxidants and total oxidants; although generally reflective of the same trends, they occasionally may lead to different conclusions. As a result, when studying the changes of oxidative status, the use of at least two or three different assays for estimation of total serum oxidants and total serum antioxidants is advisable. The application of the oxidative stress and antioxidant status in large-scale studies and automation of the commercial assays on clinical autoanalyzers is a prerequisite. Automation has been successfully applied for the assays ROM, TOS, BAP, TAS and TTP. The use of these assays in large epidemiological studies, directed to nutrition and chronic diseases, will contribute substantially to understanding the real importance of the assays.

## Figures and Tables

**Table 1:** The mean values ± SD and coefficient of variation (CV) (inter-individual) of the various assays in serum samples of 43 volunteers.

Assay	Mean ± SD	CV (%)
FRAP (μmol/L)	1,392 ± 158	11.4
BAP (μEq/L)	2,455 ± 147	6.0
TAS (μmol/L)	1,570 ± 140	8.9
TTP (μmol/L)	431 ± 52	12.1
OXY (μmol HClO/mL)	416 ± 62	14.9
Uric acid (μmol/L)	310 ± 52	16.8
ROM (CARR U)	396 ± 85	21.5
TOS (μmol H_2_O_2_ Eq/L)	1.99 ± 0.85	42.7

FRAP—ferric reducing ability of plasma; BAP—biological antioxidant potential; TAS—total antioxidant status; TOS—total oxidant status; TTP—total thiols in proteins; OXY—oxy-adsorbent test.

**Table 2:** Coefficients of correlation between the assays as performed on serum samples of 43 healthy individuals.

	FRAP	BAP	TAS	TTP	OXY	Uric acid	ROM	TOS
FRAP	--	0.097	0.807*	−0.008	0.102	0.869*	0.090	0.039
BAP		--	0.501*	0.510*	0.009	0.302	−0.037	−0.140
TAS			--	0.279	0.287	0.922*	−0.050	−0.105
TTP				--	0.042	0.114	−0.171	−0.048
OXY					--	0.272	−0.144	0.073
Uric acid						--	−0.005	−0.109
ROM							--	0.204
TOS								--

* *p* < 0.05 if the absolute value of the coefficient of correlation is higher than 0.304.
